# The impact of adding cost information to a conversation aid to support shared decision making about low‐risk prostate cancer treatment: Results of a stepped‐wedge cluster randomised trial

**DOI:** 10.1111/hex.13810

**Published:** 2023-07-02

**Authors:** Mary C. Politi, Rachel C. Forcino, Katelyn Parrish, Marie‐Anne Durand, A. James O'Malley, Rachel Moses, Krista Cooksey, Glyn Elwyn

**Affiliations:** ^1^ Department of Surgery, Division of Public Health Sciences Washington University School of Medicine St. Louis Missouri USA; ^2^ Geisel School of Medicine at Dartmouth, The Dartmouth Institute for Health Policy and Clinical Practice Dartmouth College Lebanon New Hampshire USA; ^3^ Université Toulouse III Paul Sabatier Toulouse France; ^4^ Department of Biomedical Data Science Geisel School of Medicine at Dartmouth, Dartmouth College Lebanon New Hampshire USA; ^5^ Section of Urology, Department of Surgery Dartmouth‐Hitchcock Medical Center Lebanon New Hampshire USA

**Keywords:** conversation aid, costs of care, decision aid, financial toxicity, stepped‐wedge cluster randomised design

## Abstract

**Background:**

Decision aids help patients consider the benefits and drawbacks of care options but rarely include cost information. We assessed the impact of a conversation‐based decision aid containing information about low‐risk prostate cancer management options and their relative costs.

**Methods:**

We conducted a stepped‐wedge cluster randomised trial in outpatient urology practices within a US‐based academic medical center. We randomised five clinicians to four intervention sequences and enroled patients newly diagnosed with low‐risk prostate cancer. Primary patient‐reported outcomes collected postvisit included the frequency of cost conversations and referrals to address costs. Other patient‐reported outcomes included: decisional conflict postvisit and at 3 months, decision regret at 3 months, shared decision‐making postvisit, financial toxicity postvisit and at 3 months. Clinicians reported their attitudes about shared decision‐making pre‐ and poststudy, and the intervention's feasibility and acceptability. We used hierarchical regression analysis to assess patient outcomes. The clinician was included as a random effect; fixed effects included education, employment, telehealth versus in‐person visit, visit date, and enrolment period.

**Results:**

Between April 2020 and March 2022, we screened 513 patients, contacted 217 eligible patients, and enroled 117/217 (54%) (51 in usual care, 66 in the intervention group). In adjusted analyses, the intervention was not associated with cost conversations (*β* = .82, *p* = .27), referrals to cost‐related resources (*β* = −0.36, *p* = .81), shared decision‐making (*β* = −0.79, *p* = .32), decisional conflict postvisit (*β* = −0.34, *p*= .70), or at follow‐up (*β* = −2.19, *p* = .16), decision regret at follow‐up (*β* = −9.76, *p* = .11), or financial toxicity postvisit (*β* = −1.32, *p* = .63) or at follow‐up (*β* = −2.41, *p* = .23). Most clinicians and patients had positive attitudes about the intervention and shared decision‐making. In exploratory unadjusted analyses, patients in the intervention group experienced more transient indecision (*p* < .02) suggesting increased deliberation between visit and follow‐up.

**Discussion:**

Despite enthusiasm from clinicians, the intervention was not significantly associated with hypothesised outcomes, though we were unable to robustly test outcomes due to recruitment challenges. Recruitment at the start of the COVID‐19 pandemic impacted eligibility, sample size/power, study procedures, and increased telehealth visits and financial worry, independent of the intervention. Future work should explore ways to support shared decision‐making, cost conversations, and choice deliberation with a larger sample. Such work could involve additional members of the care team, and consider the detail, quality, and timing of addressing these issues.

**Patient or Public Contribution:**

Patients and clinicians were engaged as stakeholder advisors meeting monthly throughout the duration of the project to advise on the study design, measures selected, data interpretation, and dissemination of study findings.

## 1 INTRODUCTION

Healthcare costs are rising substantially in the United States and internationally,^1^, ^2^, ^3^, ^4^, ^5^, ^6^, ^7^ leading to psychological, social, behavioural, and health‐related challenges for patients. The associated cost‐related hardship, often called *financial toxicity*, can result in delayed or forgone care^8^, ^9^ and is even associated with an increased risk of mortality.^10^, ^11^ In addition to the direct costs of healthcare, patients experience indirect cost burdens such as lost wages from missing work for appointments or illness‐related disability. Communicating the relative or specific costs of options is an important part of patient‐centred decision‐making.^12^ Patients want to know about cost information^13^, ^14^ and clinicians acknowledge its importance and impact on patients' choices and adherence to care.^15^ Yet clinician communication and patient decision aids rarely include costs of treatment options to support decisions.^16^

Although financial toxicity affects patients with cancer worldwide,^1^, ^2^, ^3^, ^4^, ^5^ it is particularly a problem for patients in the United States. The United States has a healthcare system that requires patients to use insurance to share the cost of care, and each insurance option in the private sector can vary in terms of the amount of cost‐sharing provided.^6^, ^7^ In addition, more than 30 million people in the United States are uninsured and struggle to identify ways to pay for care through hospital billing options, the government, or social service agencies.^17^, ^18^, ^19^ As many as 18% of patients in the United States have medical debt as a result,^19^ and it is the most common form of debt in the United States.^20^ Even patients who have adequate insurance coverage for cancer treatment receive out‐of‐pocket bills for copayments, medications, and support services. They also have indirect costs of care from lost wages or time off work, payment for transportation to/from health visits, and disruption to their daily routine.

In the context of early‐stage, favourable risk prostate cancer,^21^ there are several reasonable treatment options for patients to consider including active surveillance, radiation (external beam or brachytherapy) and prostatectomy (typically robotic). Each choice is similarly effective in preventing prostate cancer‐related mortality but carries different tradeoffs and costs,^22^ especially to patients with varying insurance coverage.^23^ For example, prostatectomy and radiation therapy cost more than active surveillance both in terms of out‐of‐pocket health expenses and downstream indirect costs from time off work and recovery.^22^, ^23^, ^24^, ^25^ However, some patients prefer to intervene with surgery or radiation rather than actively monitor a known cancer, even if it is low‐risk or favourable risk, because they worry about the cancer growing or spreading.^26^ Others might choose active surveillance even with the increased risk of repeat biopsies and imaging because they want to avoid the possible side effects of surgery or radiation such as incontinence and erectile dysfunction.^27^, ^28^ The complexity of trade‐offs, preferences, and difficulty estimating costs to patients can complicate shared decision‐making.

We previously developed a conversation‐based decision aid (called an Option Grid [OG]) containing information about low‐risk prostate cancer management options. OGs are brief tabular comparisons of options that activate patients before clinical visits and facilitate efficient conversations during visits.^29^ They can increase shared decision‐making across diverse contexts.^30^, ^31^, ^32^ by promoting deliberation and dialogue, while providing evidence‐based information.^33^ We added a prompt to consider the relative costs of prostate cancer management options. In past work, this approach increased the frequency of cost conversations about early‐stage breast cancer decisions.^34^ No such intervention has been developed and evaluated for prostate cancer.

In this study, we aimed to assess the impact of a conversation‐based decision aid (OG) containing cost information about low‐risk prostate cancer management options, combined with a brief training session for urologic surgeons, on the frequency and quality of patient‐urologic surgeon cost conversations. We hypothesised that:

1.1: Urologic surgeons assigned to training and use of the decision aid would engage in more frequent cost conversations than urologic surgeons in usual care.

1.2: Urologic surgeons assigned to training and use of the decision aid would be more likely to make a referral (e.g., to social service organisations, billing representatives, social workers or financial navigators) to address specific cost details than urologic surgeons in usual care.

1.3 (Exploratory): Patients of urologic surgeons assigned to training and use of the decision aid would have lower financial toxicity at 3 months follow‐up than patients of urologic surgeons in usual care.

We also aimed to examine the impact of the conversation‐based decision aid and surgeon training on decision quality, including measures of decisional conflict, decision regret, and shared decision‐making. We hypothesised that:

2.1: Patients of urologic surgeons assigned to training and use of the decision aid would report less decisional conflict, less decisional regret at 3 months follow‐up, and more shared decision‐making than patients in usual care.

## 2 METHODS

Detailed methods are described in a published protocol.^35^ Reporting follows the 2018 CONSORT extension for stepped wedge randomised controlled trials.^36^

### 2.1 Design

**Figure 1:**
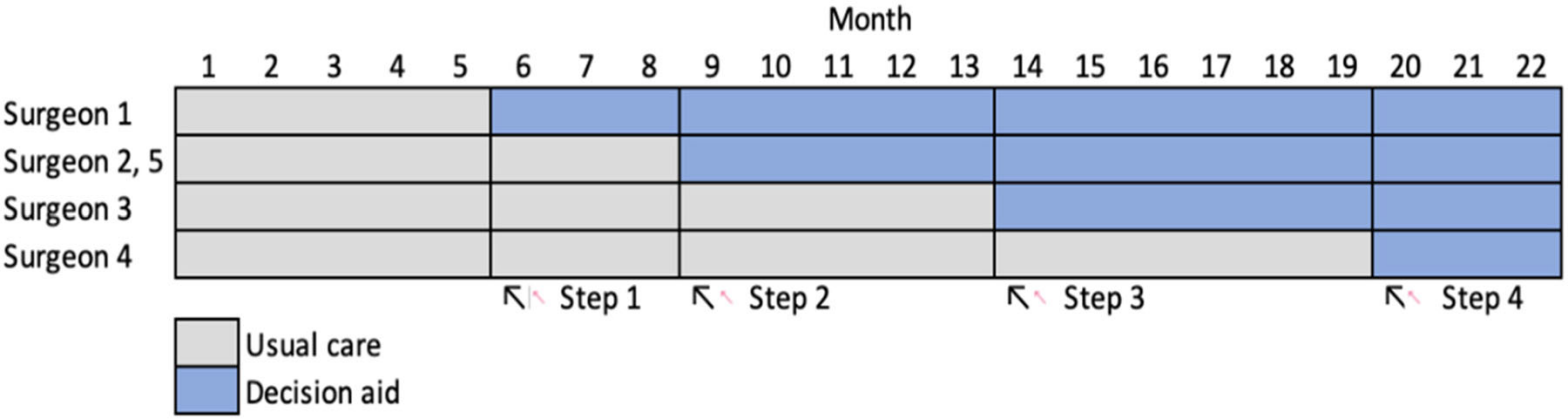
Stepped wedge design.

We conducted a stepped‐wedge cluster randomised controlled trial with urologic surgeons as clusters, four sequences, and at least one cluster assigned to each sequence (Figure 1). A stepped‐wedge design involves delivering an intervention at regular intervals, or steps, following a baseline period with no intervention. In this type of design, studies often need fewer clusters to achieve the same statistical power as a larger cluster randomised trial, and each can act as their own control due to the baseline period.^37^ In addition, because of the learning effects of a clinician‐focused intervention such as a conversation‐based decision aid, the stepped‐wedge design limits contamination in the control group. Five 3‐month periods were planned; we extended the length of some periods after trial initiation due to few eligible patients during the onset of the COVID‐19 pandemic, resulting in five periods of 5, 3, 5, 6, and 3 months, respectively. We enroled independent eligible patients in each period.

### 2.2 Settings

We conducted the study in outpatient urology clinics affiliated with a large academic medical center in the Midwest region of the United States.

### 2.3 Participants

We included five urologic surgeons who practiced at a participating clinic and routinely discussed management options for low‐risk prostate cancer with patients.

We included English‐speaking adult patients scheduled to visit a participating urologic surgeon to discuss a new diagnosis of low‐risk prostate cancer. Eligible patients had at least one of the following: (1) a Gleason score of 6 or 7 (3 + 4); (2) a prostate‐specific antigen level less than 10 ng/mL; (3) a surgeon's referral for study eligibility. We excluded patients unable to give informed consent due to cognitive or emotional barriers and those discussing recurrent or ongoing prostate cancer management. A research coordinator screened potentially eligible patients based on inclusion criteria and confirmed eligibility with the surgeon before arranging to contact the patient to gain consent to enrol in the study; surgeons could also refer patients directly to the study if they used the intervention with a patient during clinical care.

### 2.4 Patient and stakeholder engagement

At the start of the study, a patient and stakeholder advisory board was formed consisting of a survivor of prostate cancer, a patient advocate and leader of a local prostate cancer advocacy and education group, a urologist, a community engagement leader at the cancer center and expert on prostate cancer disparities, and an oncologist with expertise in financial toxicity. This team met monthly throughout the duration of the project to advise on the study design, intervention adaptation to include cost‐related information, outcome measures selected, social service organisations and personnel for cost‐related referrals, data interpretation, and dissemination of study findings.

### 2.5 Intervention and comparator

The intervention comprised an OG conversation‐based decision aid comparing management options for low‐risk prostate cancer, with relative cost information included for each option in addition to referral information for general and local resources for navigating care costs (Appendix A). OGs are brief, tabular comparisons of options written at an accessible reading level and organised by common patient questions.^29^ OGs are used collaboratively by clinicians and patients to facilitate conversations and decision dialogue, while providing evidence‐based information.^38^ They are particularly useful in situations when patients might not have had time to prepare for a decision discussion, such as low‐risk prostate cancer when patients often receive biopsy results from a clinician immediately before discussing management options. At the study start, we merged previously developed and tested OG information into a research version of an OG to compare active surveillance, surgical treatment and radiation treatment for low‐risk prostate cancer. The cost information was added to the common questions about trade‐offs between options and was generated from the literature available at the time of the study start.^24^, ^25^, ^39^ To display comparative cost‐related information, we included a visual icon to represent relative costs to patients ($–$$, $$–$$$, $$$) across options. We reviewed intervention adaptations with our stakeholder advisors, clinical partners, and study team members for clarity, feasibility, acceptability and appropriateness, and tracked adaptations systematically based on standards in implementation science.^40^ Future work is ongoing to quantify more precise cost‐level estimates across treatment options, but in this work, we encouraged clinicians and patients to weigh relative costs, and then refer patients to discuss more precise, personalised costs or resources with social workers, financial navigators, billing specialists or social service organisations. The study coordinator (K. P.), clinician, or administrative clinic personnel delivered the intervention to individual patients before or during a first discussion of management options following a new diagnosis of low‐risk prostate cancer as defined above. After trial initiation, we adapted to allow clinicians who forgot to introduce the intervention before or during the initial patient visit to send the intervention to a patient postvisit, tracking adaptations using a standard framework.^40^ The comparator was usual care.

Before study initiation, participating clinicians were trained in the study protocol. At the step initiating their entry into the intervention arm, each clinician attended a 30 min virtual training session in shared decision‐making, use of the intervention, and cost‐related resource and referral information.

### 2.6 Outcomes

#### 2.6.1 Primary outcomes

We measured patient reports of cost conversations and whether or not a referral was made to discuss costs in a questionnaire collected immediately postvisit (T1).

Soon after trial initiation and before any observational data were collected, we discontinued planned observational data collection using an observer‐reported cost conversation checklist.^41^ This change occurred because of the COVID‐19 pandemic which prompted mostly telehealth visits at participating clinics during the early months of the study and compromised the feasibility of having study team members audio record clinic visits in person for any in‐person consultations. We attempted to have clinicians record the consultations using the telehealth software, but the new and changing software, combined with the challenges and pressures of the COVID‐19 pandemic, and patients' hesitation to be recorded in their own homes with family members often present (even if clinicians were only to save the audio recordings, the recordings had to be created with video to start if patients were having a consultation with video turned on) made this process cumbersome. Instead, we decided to rely on the patient‐reported questionnaire to simplify the process for all stakeholders.

#### 2.6.2 Secondary outcomes

In the postvisit (T1) questionnaire, we measured patient‐reported decisional conflict (SURE^42^), shared decision‐making (collaboRATE^43^), and treatment choice preferred. We measured financial toxicity (COST^44^, ^45^) as an exploratory outcome.

At 3‐month follow‐up (T2), we measured decisional conflict (SURE^42^), decision regret (decision regret scale^46^), treatment choice received, and financial toxicity (COST^44^, ^45^) in a questionnaire distributed to participants by email or telephone.

#### 2.6.3 Exploratory implementation outcomes

Among clinician participants, we measured feasibility, appropriateness, and acceptability of sustained intervention use with the Feasibility of Implementation Measure, Appropriateness of Implementation Measure, and Acceptability of Implementation Measure.^47^ These four‐item validated measures use a five‐point ordinal scale, ranging from ‘completely disagree’ (score = 1) to ‘completely agree’ (score = 5). Higher scores indicate greater feasibility, appropriateness, and acceptability. We also measured clinicians' attitudes toward shared decision‐making and cost conversations using the ADOPT scale^48^ which lists words to describe using an intervention (e.g., CostTalk) and asks people how they feel about it.

Among patient participants, we assessed preferences for having cost conversations, with whom they prefer having cost conversations using an adapted validated measure on a five‐point ordinal scale.^14^ We assessed their confidence having cost conversations using a four‐item measure on a four‐point ordinal scale.^49^ Among patient participants who received the CostTalk intervention, we measured acceptability of the intervention using the Acceptability of Implementation Measure.^47^ These exploratory outcomes were added after study initiation.

*Sample size*. We assumed an intraclass correlation coefficient (ICC) value of 0.05 and a feasible total sample size of 200 patients across the five participating urologic surgeons. We estimated power directly for a stepped‐wedge design by solving for its value given the specified ICC of 0.05 and the within‐cluster variance of a Bernoulli (binary‐valued) random variable (conservatively assumed to be its maximum value of 0.25. We based effect size assumptions on a prior study demonstrating a significant effect when comparing the impact of a decision aid with comparative cost information to a decision aid without cost information or usual care on cost conversation frequency (66.7% vs. 33.3%).^34^ Using a two‐sided test, the power to detect a difference in our primary outcome of cost conversation frequency was estimated to be 0.804.

### 2.7 Randomisation

The principal investigator (M. P.) and study coordinator (K. P.) enroled clusters. The study statistician (A. J. O.), masked to cluster identity, generated the randomisation schedule and randomly allocated clusters (urologic surgeons) to the intervention sequences with a simple randomisation approach using R statistical software. Across the control and intervention arms, the study coordinator (K. P.) enroled consecutive eligible patients who provided informed consent to participate.

### 2.8 Statistical analysis

We first performed unadjusted bivariate comparisons of outcomes and predictors across intervention groups using *t*‐tests for continuous variables and *χ* ^2^ or Fisher's exact tests (where indicated due to small sample sizes) for categorical variables. For primary and secondary binary‐valued outcomes (e.g., cost conversations), we conducted logistic mixed‐effectss regression analysis adjusting for patient educational attainment (less than college degree vs. college degree or more), patient employment status (full‐time work vs. other), telehealth versus in‐person visit, visit date, and the binary indicator variables of the study time period when the patient began follow‐up as fixed effects, and clinician random effects to account for clustering of patient participants by urologic surgeon. We use analogous linear mixed‐effect regression models to analyse outcomes with multilevel scales (e.g., decision regret scale^46^). In both types of models, we accounted for the stepped‐wedge study design as well as the above‐mentioned patient covariates. Formerly, let $Y_{ijt}$ denote an outcome measured on the *i*th patient of the *j*th surgeon in time period *t*, ${\mathrm{OG}}_{jt}$ indicate whether surgeon *j* has transitioned from usual care to the OG by time period *t*, and $X_{ijt}$ denotes a vector of covariates on the *i*th patient of the *j*th surgeon in time period *t*. The logistic and linear mixed‐effect regression using models have the general form:

(1) $$\text{logit}(\text{Pr}(Y_{ijt}=1|\theta_{j}))=\beta_{0}+\beta_{1}{\mathrm{OG}}_{jt}+\beta_{2}X_{ijt}+\lambda_{t}+\theta_{j},$$

and

(2) $$Y_{ijt}=\beta_{0}+\beta_{1}{\mathrm{OG}}_{jt}+\beta_{2}X_{ijt}+\lambda_{t}+\theta_{j}+\varepsilon_{ijt},$$

where in both models ${\left\{\lambda_{t}\right\}}_{t=2:5}$ adjusts for time periods 2 through 5 (time period 1 is the baseline period) and $\theta_{j}$ is a random effect specific to surgeon *j* assumed to be drawn from a normal distribution with mean 0 and an unknown variance. For the linear regression model only, $\in_{ijt}$ is an idiosyncratic error term assumed to be drawn from a normal distribution with mean 0 and an unknown variance. In checking for outliers, we identified a few erroneously coded variables that we corrected. While we were prepared to use multiple imputation methods if the missing data was extensive, because drop‐out only occurred at time period 2 (i.e., there was no dropout up to time period 1) and most outcomes were analysed at time period 1, we favoured the use of complete‐case analyses for all analyses.

## 3 RESULTS

**Figure 2:**
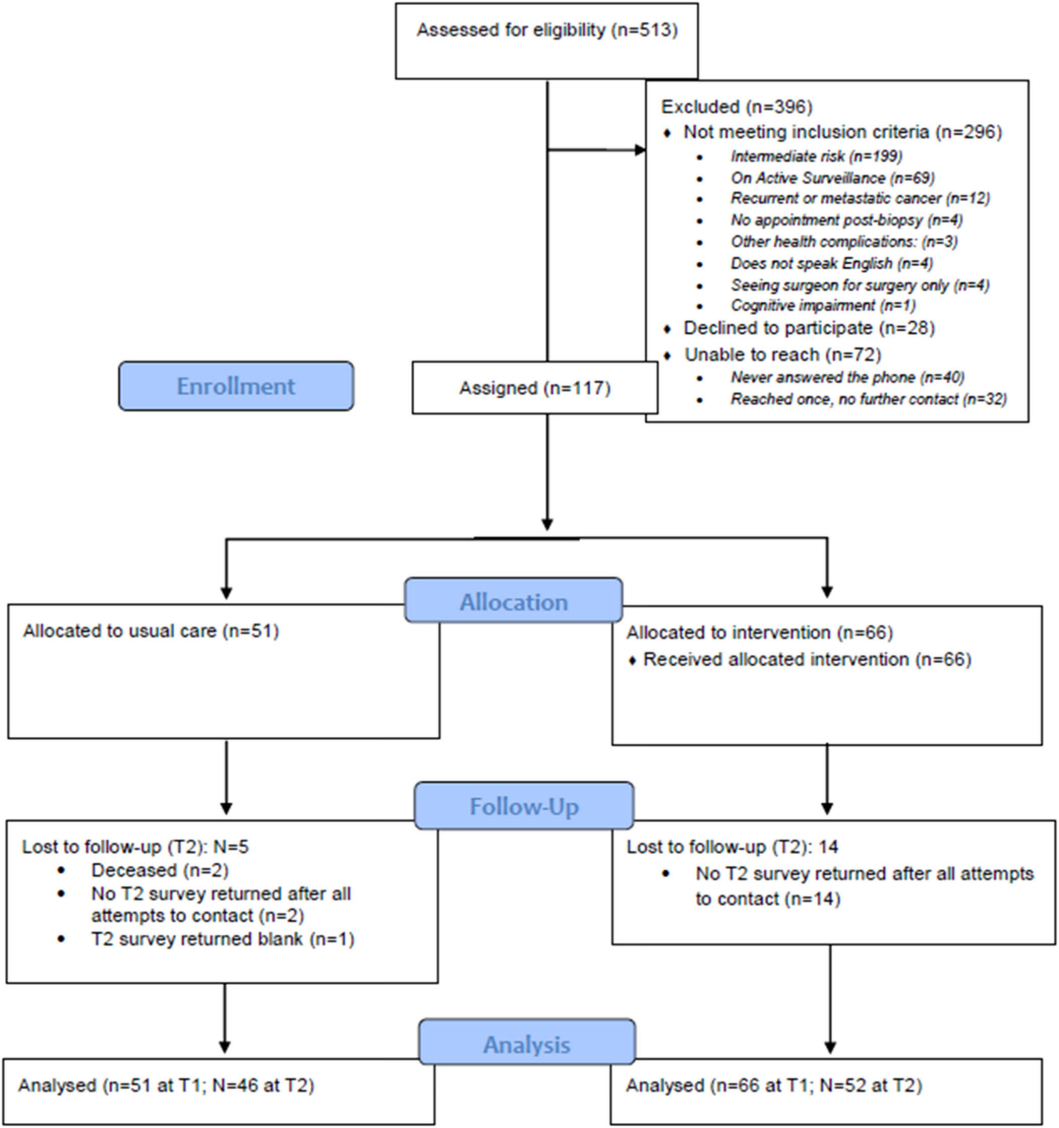
CONSORT flow diagram.

*Participant flow*: Between April 2020 and March 2022, 513 patients were assessed for eligibility. 296 did not meet inclusion criteria; 79 had a Gleason 6 or 7 intermediate risk, 120 had a Gleason score greater than or equal to 8, 69 were on active surveillance, 6 had recurrent cancer, 6 had metastatic cancer, 4 did not have an appointment postbiopsy, 3 had additional health complications, 4 did not speak English, 4 were seeing the surgeon for surgery only, and 1 patient was cognitively impaired. A total of 28 patients declined to participate and 72 patients were unable to be reached (40 patients never answered the phone and 32 patients were reached once with no additional contact). Of the 217 eligible patients that were contacted, 117 provided informed consent to participate. Figure 2 shows participant flow details.

### 3.1 Patient participant characteristics

We enroled 117 patients total, of which 51 were randomised to usual care and 66 to the intervention condition. Most participants were White (84%), non‐Hispanic (88%), and had a college degree or more education (56%). Reflecting the onset and progression of the COVID‐19 pandemic, 49% of patient visits in the usual care arm were conducted via telehealth; 15% of patient visits in the intervention arm were telehealth. Table 1 displays participant characteristics.

**Table 1:** Enroled patient participant characteristics.

	Total (*n* = 117)	Usual care (*n* = 51)	Intervention (*n* = 66)	Unadjusted *p*‐value
Age				.41
Mean	63.1 (SD: 7.7)	62.4 (SD: 7.5)	63.6 (SD: 7.9)	
Range	39–78	46–77	39–78	
Race				.11
White	98 (84%)	42 (82%)	56 (85%)	
Black	8 (7%)	6 (12%)	2 (3%)	
Another race or more than one race	11 (9%)	3 (6%)	8 (12%)	
Ethnicity				.19
Latino/a/x or Hispanic	2 (2%)	0 (0%)	2 (3%)	
Not Latino/a/x or Hispanic	103 (88%)	48 (94%)	55 (83%)	
Missing	12 (10%)	3 (6%)	9 (14%)	
Education				.06
Less than college degree	42 (36%)	14 (27%)	28 (42%)	
College degree or more	66 (56%)	34 (67%)	32 (48%)	
No response	10 (8%)	3 (6%)	7 (10%)	
Household income	(*N* = 89)	(*N* = 42)	(*N* = 47)	.16
Median (IQR)	$100K ($88K)	$100K ($97K)	$100K ($82.5K)	
Range	$16K–$1.5M	$20K–$1.5M	$16K–$350K	
Employment status				.09
Full time paid work	52 (44%)	25 (49%)	27 (41%)	
Part time paid work	10 (9%)	3 (6%)	7 (11%)	
Unemployed	6 (5%)	5 (10%)	1 (2%)	
Retired	41 (35%)	18 (35%)	23 (35%)	
Other	4 (3%)	0 (0%)	4 (6%)	
Missing	4 (3%)	0 (0%)	4 (6%)	
Surgeon				.01
A	8 (7%)	2 (4%)	6 (9%)	
B	50 (42%)	18 (35%)	32 (48%)	
C	6 (5%)	6 (11%)	0 (0%)	
D	14 (12%)	9 (18%)	5 (7%)	
E	39 (33%)	16 (32%)	23 (35%)	
Type of visit				<.01
In‐person	83 (70%)	26 (51%)	57 (85%)	
Telemedicine	35 (30%)	25 (49%)	10 (15%)	
Other common health conditions				.64
1 Or more	103 (88%)	45 (88%)	58 (88%)	
None	11 (9%)	4 (8%)	7 (11%)	
Missing	3 (3%)	2 (4%)	1 (2%)	
Option Grid delivery				–
Before visit	–	–	14 (21%)	
During visit	–	–	41 (61%)	
After visit	–	–	11 (16%)	

Abbreviation: IQR, interquartile range.

### 3.2 Aim 1: Cost conversation outcomes

Table 2 presents summary statistics for the cost conversation outcomes at postvisit (T1). Full regression results are reported in Appendix C.

**Table 2:** Postvisit (T1) cost conversation summary statistics.

	Usual care	Intervention	Adjusted *p*‐value
	(*n* = 51)	(*n* = 66)	
Investigator‐coded response: Were any cost topics discussed?			.27
Yes	47.1% (24)	43.9% (29)	
Insurance eligibility or coverage	23.5% (12)	25.7% (17)	
Time off work during treatment or recovery	19.6% (10)	15.2% (10)	
Cost of specific tests, or visits, or equipment	11.8% (6)	12.1% (8)	
Comparing costs of options to help choose care	5.9% (3)	6.1% (4)	
Cost of over‐the‐counter drugs or supportive care drugs, like those to relieve side effects	2.0% (1)	3.0% (2)	
Other	2.0% (1)	3.0% (2)	
Cost of drugs to treat your cancer	0% (0)	3.0% (2)	
Cost of getting to and from healthcare visits	0% (0)	3.0% (2)	
Costs of day‐to‐day living	0% (0)	0% (0)	
Investigator‐coded response: Were any cost strategies discussed?			.49
Yes	15.7% (8)	18.2% (12)	
What cost strategies were discussed?			
Changing logistics of care	7.8% (4)	6.1% (4)	
Choosing a lower cost procedure or scan	5.9% (3)	0% (0)	
Choosing a generic instead of a brand name drug	3.9% (2)	4.6% (3)	
Copay assistance, coupons, rebates, or samples	3.9% (2)	4.6% (3)	
Referring to hospital billing office	3.9% (2)	3.0% (2)	
Setting up a payment plan for bills	2.0% (1)	4.6% (3)	
Suggesting government or VA assistance	2.0% (1)	3.0% (2)	
Changing the dose (amount) of treatments, or how often you got treatments, or how the treatments were given (such as a pill instead of an infusion)	2.0% (1)	0% (0)	
Suggesting that you talk to human resources about your time‐off or insurance benefits	2.0% (1)	0% (0)	
Suggesting a free drug programme	0% (0)	1.5% (1)	
Other	0% (0)	1.5% (1)	
Referral made to learn about costs			.81
Yes	5.9% (3)	7.6% (5)	
COST financial toxicity	*n* = 45	*n* = 57	.97
Mean	10.9 (SD: 7.7)	11.1 (SD: 8.1)	
Median	9	8	
Range (possible range 0–44)	0–34	0–34	

*Cost conversation frequency*: Unadjusted comparisons showed no increase in cost conversation frequency between usual care (47.1%) and intervention (43.9%) arms (*χ* ^2^ = 0.11, *p* = .74). In adjusted logistic regression analysis, the intervention was not significantly associated with more frequent cost conversations (*β* = .82, odds ratio [OR] = 2.27, *p* = .27).

*Cost conversation referrals*: In unadjusted comparisons, there were no significant differences in rates of referral to cost‐related resources between usual care (5.9%) and intervention (7.6%) arms (*χ* ^2^ = 0.22, *p* = .90). In adjusted logistic regression analysis, the intervention was not significantly associated with referrals to cost‐related resources (*β* = −.36, OR = 0.70, *p* = .81).

*Financial toxicity*: Financial toxicity, defined as the material and psychosocial burden of care costs on patients, was measured using a validated scale from 0 to 44, with higher scores representing more financial toxicity. It was consistent across usual care and intervention arms and across timepoints. Mean postvisit (T1) financial toxicity scores were 10.9 (SD: 7.7) in usual care and 11.1 (SD: 8.1) in the intervention arm. In adjusted linear regression analysis, the intervention was not significantly associated with financial toxicity scores postvisit (*β* = −1.32, *p* = .63). Financial toxicity scores at 3‐month follow‐up (T2) averaged 10.9 (SD: 9.2) in usual care compared to 10.5 (SD: 7.9) in the intervention. In adjusted linear regression analysis, the intervention was not significantly associated with financial toxicity at follow‐up (*β* = −2.41, *p* = .23).

### 3.3 Aim 2: Decision outcomes

Table 3 summarises decision‐related outcomes collected postvisit (T1). Table 4 summarises outcomes collected at 3‐month follow‐up (T2). Full regression results are reported in Appendix D.

**Table 3:** Postvisit (T1) decision outcome summary statistics.

	Usual care (*n* = 51)	Intervention (*n* = 66)	Adjusted *p*‐value
Treatment choice preferred			
Monitor with tests (active surveillance)	25.5% (13)	15.2% (10)	
Surgery	45.1% (23)	31.8% (21)	
Radiation	2.0% (1)	6.1% (4)	
Other (cryotherapy or cryoablation)	3.9% (2)	3.0% (2)	
Not yet decided	23.5% (12)	39.4% (26)	.43
CollaboRATE	*n* = 48	*n* = 61	.32
Top box	52.9% (27)	54.5% (36)	
Communication preferences^a^—% *Agree* or *Strongly Agree*		*n* = 51	
I would like my doctor to talk with me about my out‐of‐pocket costs when s/he recommends a test or treatment.		66.7% (34)	.80
I would prefer to talk about the cost of my care with someone other than my doctor, such as a nurse, social worker, or financial counsellor.		27.5% (14)	
I prefer to know about the out‐of‐pocket costs for my treatment before I am treated.		74% (37)	
My doctor should consider my out‐of‐pocket costs as s/he makes a medical decision.		31.4% (16)	
I consider my out‐of‐pocket costs when I make a decision about my care.		25.5% (13)	
SURE	*n* = 48	*n* = 62	.70
Decisional conflict	25.0% (12)	29.0% (18)	
No decisional conflict	75.0% (36)	71.0% (44)	

^a^ Measure was added after study initiation.

**Table 4:** 3‐Month follow‐up (T2) summary statistics.

	Usual care 3 months follow‐up (*n* = 46)	Intervention 3 months follow‐up (*n* = 52)	Adjusted *p*‐value
Treatment choice received			
Monitor with tests (active surveillance)	34.8% (16)	26.9% (14)	
Surgery	50.0% (23)	69.2% (36)	
Radiation	10.9% (5)	7.7% (4)	
Other (cryotherapy or cryoablation)	2.2% (1)	–	
Not yet decided	8.7% (4)	1.9% (1)	.62
SURE	*n* = 46	*n* = 51	.16
Decisional conflict	17.4% (8)	9.8% (5)	
No decisional conflict	82.6% (38)	90.2% (46)	
Decision regret	*n* = 46	*n* = 50	.11
Mean (SD)	12.6 (SD: 17.5)	11.2 (SD: 13.8)	
Median	2.5	5	
Range (possible range 0–100)	0–50	0–50	
COST financial toxicity	*n* = 44	*n*=48	.23
Mean	10.9 (SD: 9.2)	10.5 (SD: 7.9)	
Median	9	8	
Range (possible range 0–44)	0–36	0–36	

*Shared decision‐making*: Unadjusted comparisons of collaboRATE shared decision‐making scores showed no significant differences between usual care and intervention arms. In usual care, 52.9% of patients reported top box shared decision‐making scores compared to 54.5% of patients in the intervention arm (*χ* ^2^ = 0.03, *p* = .87). In adjusted logistic regression analysis, the intervention was not significantly associated with shared decision‐making scores (*β* = −.79, OR = 0.45, *p* = .32).

**Figure 3:**
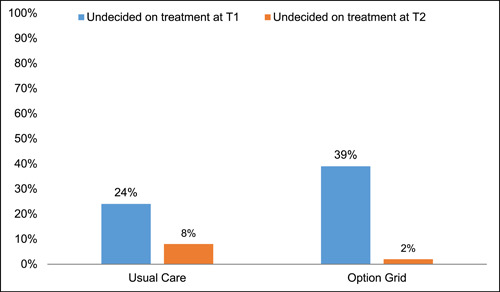
Treatment indecision and transient uncertainty at T1 that resolved by T2.

*Treatment choice and deliberation*: In unadjusted analyses, participants in the intervention arm appeared less likely to have decided on a treatment plan postvisit (T1) than participants in usual care (39.4% vs. 23.5% undecided; *χ* ^2^ = 3.30, *p* = .07; Figure 3). This reversed at 3 months follow‐up (T2), where participants in the intervention arm appeared slightly more likely to have decided on a treatment plan than participants in usual care (1.9% vs. 8.7% undecided; *χ* ^2^ = 2.82, *p* = .09; Figure 3). In adjusted logistic regression analysis of those undecided versus decided on any treatment, the intervention was not significantly associated with treatment indecision postvisit (*β* = .64, OR = 1.90, *p* = .43) or at 3 months follow‐up (*β* = −1.39, OR = 0.25, *p* = .62). However, among those undecided on treatment at T1 and who had not decided on treatment by T1 (usual care *n* = 12, OG *n* = 26), the OG was associated with improved resolution of treatment indecision by 3 months follow‐up compared to usual care (unadjusted Fisher's exact test, *p* < .03). Due to the small sample size available for this analysis (*n* = 38) and the high conversion of patients to treatment resolution in the intervention arm, our ability to estimate models that adjusted for study time‐period, other patient covariates, and physician random‐effects was compromised with the effects of most such variables being inestimable. However, the one variable whose effect could be adjusted for was visit date and we found minimal evidence that it was associated with the resolution of treatment indecision.

*Decisional conflict*: At T1, 25.0% of participants in usual care and 29.0% of participants in the intervention arm reported decisional conflict immediately postvisit (*χ* ^2^ = 0.22, *p* = .64). At T2 3 months later, 17.4% of participants in usual care and 9.8% of participants in the intervention arm reported decisional conflict (*χ* ^2^ = 1.20, *p* = .27). In adjusted logistic regression analyses, the intervention was not significantly associated with decisional conflict postvisit (*β* = −.34, *p* = .70) or at 3 months follow‐up (*β* = −2.19, *p* = .16).

*Decision regret*: In unadjusted comparisons of the decision regret score (0–100 scale), there were no significant differences in decision regret reported in usual care (*m* = 12.6, SD = 17.5) and intervention (*m* = 11.2, SD = 13.8) arms at 3 months follow‐up (*t* = 0.44, *p* = .66). Adjusted linear regression analysis showed no significant association between the intervention and decision regret scores (*β* = −9.76, *p* = .11).

### 3.4 Implementation outcomes

Most clinicians had positive views on using the intervention. Regarding acceptability, all clinicians stated that they approved of the intervention (100% agreed or strongly agreed; mean = 4.2 [SD: 0.4], range: 4–5), and 4/5 (80%) said they welcomed using the intervention in practice moving forward (mean = 3.8 [SD: 0.4], range: 3–4). The majority of clinicians found the intervention fitting, suitable, and applicable for their practice (80% agreed or completely agreed; mean = 3.8 [SD: 0.4], range: 3–4), highlighting the appropriateness. Regarding feasibility, 80% agreed or completely agreed that it seems implementable in their practice and seems easy to use. However, a little more than half (3/5 or 60%) of clinicians described using it as easy and effective, only 2/5 (40%) described it as timesaving and collaborative, and 1/5 (20%) described it as necessary. One clinician (20%) described it as inefficient and laborious.

Patients stated that they preferred having cost discussions with a doctor (68% agreed or strongly agreed; mean = 3.9 [SD: 1.0], range: 2–5), compared with a social worker or financial counsellor (only 28% agreed or strongly agreed; mean = 3.0 [SD: 1.1], range: 1–5). Additionally, patients wanted to know their out‐of‐pocket costs before they were treated (75% agreed or strongly agreed; mean = 4.0 [SD: 1.0], range: 2–5), but were mixed in terms of whether they consider out‐of‐pocket costs when making treatment decisions (28% agreed or strongly agreed; mean = 2.8 [SD: 1.2]).

The majority of patients who received the intervention approved of their doctor using the intervention (70% agreed or strongly agreed; mean = 3.9 [SD: 0.7], range: 3–5). Additionally, most patients found the intervention appealing and welcomed their doctor using it (mean = 3.7 [SD: 0.8], range 2–5 and mean = 3.8 [SD: 0.8], 3–5, respectively).

## 4 DISCUSSION

This study evaluated an intervention to improve cost discussions between urologic surgeons and patients when deciding how to manage low‐risk prostate cancer. Most decision aids and decision aid standards do not include cost information,^9^, ^50^ even though patients report that costs impact their choices and the implementation of those choices.^12^, ^51^, ^52^ Despite extensive engagement with the clinical teams and high enthusiasm from both clinicians and patients using the intervention, the intervention was not significantly associated with the hypothesised outcomes, though we were unable to robustly test outcomes due to recruitment challenges. There were early indicators of a positive association between the intervention and cost conversation frequency in adjusted analyses, though these results were not statistically significant. In addition, it appeared that the decision aid‐based intervention supported active deliberation based on the number of people undecided after their appointment, which resolved at their 3 months follow‐up; this analysis was supported by a small sample size and did not allow for a fully adjusted model to be estimated, thus is presented as an exploratory finding worthy of future study. When adjusting for patient education, employment, telehealth versus in‐person visit, visit date, and the enrolment time period as fixed effects, and clinician as random effects, results did not show significant associations with cost conversations or decision outcomes.

It is possible that those enroled in the study early in the recruitment period had higher financial toxicity and financial uncertainty due to the onset of the COVID‐19 pandemic. We did note that more participants early in the study reported lower incomes and higher financial toxicity scores; these challenges were faced by many individuals in 2020 as jobs required individuals to stay home to avoid spreading illness, and the economy suffered from widespread shutdowns which were necessary but placed financial strain on individuals and businesses. It is also possible that the use of telehealth (which was more common early in the study, common in 2020 for those with nonurgent needs and those who did not need in‐person care) impacted the way in which clinicians and patients discussed options and costs openly. In our study's regression analyses adjusting for these factors, neither visit type (telehealth or in‐person) nor visit date were significantly associated with our outcomes. It was important to adjust for these factors in our analyses, but we might not have been able to detect intervention effects above and beyond these differences.

In addition, it is possible that clinicians need more than a conversation‐based decision aid and brief training to encourage cost conversations. Clinicians were enthusiastic about the intervention, but it is difficult to change typical conversational flow and content. Perhaps more documented impact on patient outcomes and more in‐depth role‐playing interventions could serve as stronger motivators and increase self‐efficacy for discussing costs. Alternatively, perhaps the patients in our study were less concerned about costs because many were employed with relatively high incomes. A larger study could engage a more socioeconomically diverse group of patients.

It is interesting to note that the intervention might not have encouraged cost‐related referrals. The conversation‐based decision aid listed resources for patients to contact for assistance or questions with financial aspects of care (e.g., social workers, financial navigators, hospital billing representatives and community organisations). It also listed questions patients could ask if they wanted to learn more about their costs. Perhaps clinicians using the intervention felt that the conversation‐based decision aid covered this information without having to bring it up directly, or that many patients in this study did not need additional referrals to discuss costs.

Despite the limited impact on cost conversations and decision outcomes as measured, clinicians reported that they approved of the intervention and most wanted to use it beyond the study period. Some noted verbally that patients appreciated the intervention and even referenced it over time at subsequent visits. Clinicians provided suggestions for ways to improve the cost‐related information in the decision aid that might help future work. For example, some clinicians commented that while active surveillance may cost less to the patient up front, the cost of repeated biopsies and imaging over time adds to these costs and could make active surveillance equivalent to surgery/radiation costs in 5–10 years. In addition, although the cost to patients between surgery or radiation might be equivalent or close to it, the cost to the healthcare system might be much higher for those who choose external beam radiation. We looked into the literature to clarify these questions raised by clinicians, and the data were limited or outdated on actual costs to patients and to the healthcare system. Future studies could explore more precise costs to patients and the healthcare system over time.

Finally, the number of people in the intervention arm who deliberated about options—remaining undecided upfront and resolving their uncertainty over time—warrants further investigation. One of the main goals of shared decision‐making is to encourage patients to choose a treatment that aligns with preferences, taking the time they need to think through options. Most people in the control group (75%) made decisions upfront at the time of their appointment, while almost 40% were undecided in the intervention arm at the time of their appointment. At the 3 months follow‐up, however, this uncertainty resolved, and only 2% remained undecided in the intervention arm. Perhaps those assigned to use the OG spent more time weighing their options. Future work could explore the process of shared decision‐making and deliberation with and without a decision aid.

Strengths of the study included the highly engaged clinical team, many of whom thanked the research staff during and after the study for the intervention and resources. Some reported anecdotally that patients were bringing the intervention back to the clinic at their follow‐up visit(s) months or even a year later. In addition, adding exact costs to decision aids is resource‐intensive and often varies widely by patient. We used relative cost information and referrals to incorporate costs into an existing, previously tested conversation aid. The engagement of patient and stakeholder advisors also allowed us to adapt an intervention and develop a study flow that met the needs of end users even during difficult times such as the onset of the COVID‐19 pandemic. The intervention was rated feasible and acceptable, and some plan to continue using even beyond the study period.

However, limitations included the small sample size (of about 60% of that planned) and heterogeneity of the number of patients by clinician, which impacted our statistical power to detect differences between groups. In addition, the timing of early recruitment began during the early stages of the COVID‐19 pandemic limiting our ability to identify patients in‐person and audio‐record conversations. In addition, COVID‐related delays in seeking care could have impacted the representativeness of our sample based on our inclusion criteria that men had to have low‐risk prostate cancer. Low‐risk prostate cancer is generally less commonly diagnosed among Black men, and Black men often delayed seeking care for prostate cancer during 2020.^53^ Moreover, changes in clinical staffing led to smaller sample sizes than anticipated overall, and smaller cluster sample sizes. One clinician stopped treating patients with prostate cancer just before the clinician's randomised assignment to the intervention. This clinician often treated a more socioeconomically and racially diverse patient group at a satellite hospital facility. Across the analyses, the intervention was among the stronger of the associations despite being nonsignificant. Therefore, the sample size limitation, the heterogeneity of the number of patients by clinician, and a smaller than anticipated effect size on our primary outcome (47.1% discussing costs in usual care vs. 43.9% in intervention) affected our ability to detect differences between groups.

Future work should explore ways to engage clinicians and patients in shared decision‐making and cost conversations during or after the clinical visit. Such work could involve additional member(s) of the care team, explore the best time to address these important issues, consider the amount, specificity and quality of cost information presented, directly ask about deliberation, and record or observe visits to assess outcomes.

## AUTHOR CONTRIBUTIONS

Dr. Mary C. Politi led all aspects of the work including the design of the work, analysis and interpretation of data, and writing and revising the manuscript. Dr. Rachel C. Forcino contributed to the analysis and interpretation of data and writing and revising the manuscript. Ms. Katelyn Parrish contributed to the acquisition of the data and revising the manuscript. Dr. Marie‐Anne Durand contributed to the design of the work, analysis and interpretation of data, and revising the manuscript. Dr. A. James O'Malley led the analysis and interpretation of data, and contributed to writing and revising the manuscript. Dr. Rachel Moses contributed to the analysis and interpretation of data and revising the manuscript. Ms. Krista Cooksey contributed to the analysis of data and revising the manuscript. Dr. Glyn Elwyn co‐led the design of the work, analysis and interpretation of data, and writing and revising the manuscript. All authors have approved the final version.

## CONFLICT OF INTEREST STATEMENT

Dr. Mary C. Politi was a consultant for UCB Biopharma 2022–2023 on a topic unrelated to this manuscript. Drs. Glyn Elwyn and Marie‐Anne Durand have developed Option Grid patient decision aids, which are licensed to EBSCO Health; they receive consulting income from EBSCO Health and may receive royalties in the future.

## ETHICS STATEMENT

The study was reviewed and approved by the Washington University in St. Louis Human Research Protection Office (project #202004249). All participants provided informed consent to participate. This study was registered at ClinicalTrials.gov on 21 May 2020, (https://clinicaltrials.gov/ct2/show/NCT04397016).

## Data Availability

The datasets generated during and/or analysed during the current study are available from the corresponding author on request.

## Appendix B ADDITIONAL POSTVISIT (T1) DECISION OUTCOME SUMMARY STATISTICS

**Table:**

	Usual care (*n* = 51)	Intervention (*n* = 66)	Adjusted *p*‐value
*Consumer assessment of healthcare providers and systems (CAHPS) communication measure (%) ‘Yes, definitely’*	*n* = 48	*n* = 62	.33
During your most recent visit, did your urologist explain things in a way that was easy to understand?	92.2% (47)	90.9% (60)	
During your most recent visit, did your urologist listen carefully to you?	92.2% (47)	92.4% (61)	
During your most recent visit, did your urologist show respect for what you had to say?	94.1% (48)	92.4% (61)	
During your most recent visit, did your urologist spend enough time with you?	90.2% (46)	89.4% (59)	
*Perceived efficacy in patient–physician interactions* ^a^		*n* = 38	.85
Mean		13.8 (SD: 3.5)	
Median		16	
Range (possible range 0–16)		4–16	

^a^ Measure was added after study initiation.

## Appendix C PRIMARY OUTCOME REGRESSION RESULTS

**Table:**

	Cost conversations	Cost strategies	Cost referrals
	Coefficient	Coefficient	Coefficient
Study step 1	(Reference)	(Reference)	(Reference)
Study step 2	−0.197	0.337	−0.81
Study step 3	0.461	−24.07	−29.78
Study step 4	0.261	0.783	−0.04
Option Grid	0.822	0.620	−0.04
Education	0.048	0.169	−31.00
Employment	0.305	0.045	−1.30
Telehealth	0.643	1.269	0.87
Visit date	−0.003	−0.002	<0.01
	Variance	Variance	Variance
Clinician	<0.001	0	0

## Appendix D SECONDARY OUTCOME REGRESSION RESULTS

**Table:**

	Undecided on treatment (T1)	Undecided on treatment (T2)	CollaboRATE (T1)	SURE (T1)	Decision regret (T2)
	Coefficient	Coefficient	Coefficient	Coefficient	Coefficient
Study step 1	(Reference)	(Reference)	(Reference)	(Reference)	(Reference)
Study step 2	−0.23	19.03	0.43	0.77	−2.49
Study step 3	−0.30	−8185.0	1.12	−0.36	−6.33
Study step 4	−0.98	−4215.0	−0.02	−0.31	−10.13
Option Grid	−0.64	−1.39	−0.79	−0.34	−9.76
Education	−17.10	−2217.0	−0.24	0.54	14.66
Employment	−0.09	22.71	−0.85	−15.98	3.99
Telehealth	−0.41	−2565.0	−0.68	0.09	−8.07
Visit date	<0.01	<0.01	<0.01	<0.01	0.03
	Variance	Variance	Variance	Variance	Variance
Clinician	0.43 (SD: 0.65)	0	0	0.35 (SD: 0.59)	6.09 (SD: 2.47)
